# Key Factors Influencing the Food Choices of Athletes at two Distinct Major International Competitions

**DOI:** 10.3390/nu12040924

**Published:** 2020-03-27

**Authors:** Rachael Thurecht, Fiona Pelly

**Affiliations:** School of Health and Sport Sciences, Faculty of Science, Health, Education and Engineering, University of the Sunshine Coast, Maroochydore, Queensland 4558, Australia; fpelly@usc.edu.au

**Keywords:** performance, nutrition, food selection, food behavior, diet

## Abstract

This study aimed to identify the factors influencing the food choices of athletes at the Universiade and Commonwealth Games and explore differences in the cohort across sport, competition history and demographic characteristics. A sample of 385 athletes (*n* = 153, 2017 Universiade, Taiwan; *n* = 232, 2018 Commonwealth Games, Australia), from 69 countries and 29 sports participated in this cross-sectional observational study. Participants rated 36 items from the Athlete Food Choice Questionnaire and 11 additional items (gut comfort, doping risk, availability, location, money, convenience, time of day, hunger, medical conditions, and food allergies) on how frequently (1 never to 5 always) each influences their food choices. “Performance”, “sensory appeal”, “food and health awareness” and “weight control” were reported as most frequently, while the least were “emotional influence”, “influence of others” and “food values and beliefs”. Commonwealth Games athletes were older, more experienced and more likely to report “performance” (median = 4.33 versus 4.00, U = 20250.0, *p* = 0.012) and less likely to report “emotional influences” (median = 2.80 versus 3.20, U = 14273.0, *p* = 0.001) than Universiade athletes. Greater numbers of younger athletes were often or always influenced by available money. Athletes across all sports reported frequently considering gut comfort in their food choices. These results can inform nutrition education strategies of high-performance athletes.

## 1. Introduction

The foods athletes choose to eat can impact on their wellbeing and performance, body composition, gut comfort and fuel available for training, competition and recovery. Despite the key role diet has on health and performance, athletes may not make appropriate food choices [1,2,3]. There are a limited number of studies that have explored individual and interpersonal determinants of food choices in athletes. While there are reported similarities to the general population, there appears to be factors that are specific to athletes that are particularly related to performance [4,5,6,7,8,9]. These studies have used a range of methods to report on food choice including self-developed questionnaires, focus groups and interviews. More recently, a tool has been developed and validated to measure the ranking of determinants of food choice in athletes (Athlete Food Choice Questionnaire (AFCQ) [10]). Factors included in the survey are the nutritional attributes of the food, emotional influences, food and health awareness, the influence of others, usual eating practices, weight control, food values and beliefs, sensory appeal and performance. Other studies have also identified additional factors of convenience [4,5,6,7,8,9], financial considerations [7,8], gut comfort [1,4,5] and hunger [1]. 

In the general population, food choice may be influenced by characteristics of the cohort, including age, gender, education and income [11,12,13]. In athletes, contextual factors such as the competition environment could also have a modulating effect on how determinants of food choice are prioritised. A study that investigated the factors influencing food choices at the 2006 and 2010 Commonwealth Games found that the appearance of the food, the athlete’s stage of competition and time of day was of greater influence for those at the 2010 Delhi games, compared to the cohort from Melbourne [9]. Despite the similar nature of the events, the authors rationalized that the differences may be due to perceptions surrounding the confidence of the food supply in the different host countries [9]. The relevance to competition may also play a role in influencing food choice. A study that investigated the reasons why athletes selected foods at a single meal during the Commonwealth Games found the potential effect on sports performance as a driving factor [1]. Similarly, a qualitative study of American college hockey players reported factors relating to performance influencing food choice, more so than taste, price and convenience during the competition season, as opposed to offseason [6]. Currently food choice has been investigated both during [1,9] and outside [4,5,6,7,8] of competition settings with individual [5,6,8] and multiple [1,4,7,9] sports. To date, no study has investigated how the competition setting and the athletes’ previous experience of competition may influence determinants of food choice.

The Universiade (World University Games) is a large-scale sporting competition that is specifically for athletes who are enrolled at university (aged 17 to 28 years) [14]. Despite qualification to compete, the standard of athlete competing at this event varies from regional to international, and thus this competition is often viewed by teams as less serious than World Championships, Commonwealth or Olympic Games. This is dependent on the emphasis placed on this competition by the country of origin, the sport and the individual competitor. To date, minimal research has been conducted on athletes competing at this event. A recent study has shown that despite athletes competing at a high level of competition, their confidence and implementation of nutrition knowledge is poor, with few receiving nutrition counselling [15]. Dietary analysis of their usual home diet was reported to be inadequate in energy, carbohydrates and calcium, as well as low in serves of vegetables and dairy foods [15]. Overall, this is consistent with studies that show that university students are at higher risk of poor nutrition [16,17,18]. 

Therefore, this study aims to identify the key factors influencing the food choices of a diverse cohort of athletes, explore differences in outcomes between two events (the 2017 Universiade and the 2018 Commonwealth Games) and describe differences across sport, history of competition and other demographic characteristics. 

## 2. Materials and Methods 

### 2.1. Setting

This cross-sectional observational study was conducted at two large scale international multisport events; the 2017 29th Summer Universiade, Taipei, Taiwan (22 sports, 10,600 athletes and officials, 134 countries [14]) and the 2018 21st Commonwealth Games, Australia (18 sports and 7 para-sports, 6600 athletes and officials 71 countries [19]). At each competition, athletes and officials live in a custom-made village, with a large-scale dining hall that provides food free of charge for 20–24 h a day for the duration of the event. 

The preparation of this article adheres to the STROBE principals (STrengthening the Reporting of OBservational studies in Epidemiology) [20]. All subjects gave their informed consent for inclusion before they participated in the study. The study was conducted in accordance with the Declaration of Helsinki, and the protocol was approved by the Ethics Committee of The University of the Sunshine Coast (HREC no. 1/71/086). 

### 2.2. Measurement Instrument

Factors influencing the food choice of athletes were collected using the AFCQ [10], which has undergone initial validation testing for internal reliability, face and content validity. An additional 11 items based on the existing literature and relevant to this study were included as stand-alone factors [5,6,7,8,9]. The additional items asked about; availability, cost, convenience, eating location, doping concerns, gut comfort, hunger, the meal, busy schedule, and medical conditions and food allergies (Commonwealth Games only). The two last items were added, to better capture the diversity of athletes at the Commonwealth Games, as this competition included para-sport events. Items were presented as neutral statements and participants ranked each on a frequency scale from 1 (never) to 5 (always). For this questionnaire, food choice referred to foods and beverages. 

An open-ended question was included for participants to report any other item/s they felt influenced their food choice. Demographic questions on age, sex, sport, country representing, language, phase of competition and competition history (level of competition) were also captured. 

### 2.3. Data Collection

This study used convenience sampling to survey athletes. Questionnaires were distributed at a nutrition desk or within the dining hall by the researchers. After a brief conversation with participants to subjectively determine their English capabilities, the researcher sought verbal consent and offered the paper questionnaire. The researcher was not in the vicinity of the athletes during survey completion. In addition to verbal consent, participants were free to stop completion and not return the questionnaire if desired. All questionnaires were anonymous, and participants took 10-20 min to complete the questionnaire. Sampling took place until the closing of the dining hall on completion of the event.

### 2.4. Data Analysis

The surveys were entered into the online version via surveymonkey.com. Data analysis included the use of Microsoft Excel (2013, Microsoft Corporation) and Statistical Package for Social Sciences (SPSS) Statistical Software (version 24.0, IBM Corporation). Data from the two samples were analysed separately and combined for additional testing between demographic characteristics. The two items on medical conditions and allergies that were collected from the Commonwealth Games cohort were examined separately. 

Participant characteristics and the factors were analysed, based on median (Mdn) and interquartile range (IQR), for continuous variables and proportions for categorical variables. Countries were grouped for analysis into regions based on geographic locations and consideration of the common dietary staples and cuisine [21]. Sports were grouped according to similarities in physical demand and mode of play (i.e., team, individual, racquet, low physical demand, but high skill as informed by categories in similar previous studies [1,9]). Data from the nine factors of the AFCQ were condensed to compute a mean score for each factor. The additional items were examined individually. Participant demographic characteristics, including age, sex, sport, country, competition event, level of competition and phase of competition were tested to examine any association to food choice items using Mann–Whitney U test (“U”) and the Kruskal–Wallis ANOVA (“H”), using Bonferroni correction for categorical variables and independent t-tests for continuous variables. A Chi-squared test was used to test the distributions between categorical variables, using post hoc pairwise comparisons and Bonferroni correction. Significance was set at *p* < 0.05; cases with missing values were excluded.

Open-end responses were examined via content analysis to identify any other items influencing participants’ food choices. The responses were given initial descriptive codes, then organised according to their codes into similar categories. The categories are presented as counts and proportions [22,23]. The content analysis was conducted by the primary researcher and adjustments were made by both researchers, until all classifications and categories were agreed upon. 

## 3. Results

A final sample of 385 questionnaires were included for analysis (Universiade *n* = 153; Commonwealth Games *n* = 232). Duplicates, people less than 18 years and those who had not completed more than half of the questionnaire items, were removed from the analysis (*n* = 39). Participants represented 69 countries and 29 sports (Table 1) and reported speaking 58 different languages, with English (62%), French (5%), Spanish (4%), Chinese (3%) and Portuguese (3%) as the five highest reported languages spoken at home. The mean age of all participants was 25 +/− 7 years (range 18–71 years). The proportion of males and females across the different sports were mostly equivalent, with only skilled sports having a higher proportion of female participants (*n* = 28, 68%). There were significantly more team sport athletes from Western regions, and less weight category and power/ sprint athletes from non-Western regions (Table 1). There were no differences across regions and sports with regards to completion of the questionnaire, pre- or post-competition.

There are some differences in demographic characteristics worth noting between the two cohorts. Commonwealth Games athletes were significantly older (Mdn 25 (23–29), versus 22 (20–23) years; *z* = 9.18, *p* < 0.001), and had a higher proportion of Olympic level competitors (*n* = 136, 95%) than those from the Universiade (*n* = 7, 5%, *p* < 0.001; Fisher’s exact test). There was a higher proportion of team sport athletes (*n* = 85, 60% versus *n* = 56, 39%; X^2^ (5) = 54.85, *p* < 0.001) in the Universiade sample, and more participants from European and Middle Eastern countries (*n* = 39, 91% versus *n* = 4, 9%; X^2^ (5) = 56.63, *p* < 0.001), as is representative of the event characteristics and country participation. A higher proportion of Universiade participants (*n* = 101, 60%) completed the questionnaire after finishing their events, compared to Commonwealth Games participants (*n* = 68, 40%, *p* < 0.001 Fisher’s exact test).

The ranking of the factors influencing food choice both combined and across events, regions and sport categories is shown in Table 2. Overall, the most frequently reported factors were “performance”, “sensory appeal”, “food and health awareness” and “weight control”. Conversely, the least frequently reported were “emotional influence”, “influence of others”, and “food values” and “beliefs”. Significant differences were found between events, regions and sport categories for performance, and food and health awareness. There were also differences between events for emotional influences and the influence of others (Table 2). 

Athletes more frequently reported “food and health awareness” (Mdn 4.00 versus 3.75) and “nutritional attributes of the food” (Mdn 3.57 versus 3.29) influencing food choice prior to competition than post-competition (U = 14551.0, *p* = 0.002; and, U = 15219.5, *p* = 0.009 respectively). Males rated “performance” (Mdn 4.00 versus 4.33) and “emotional influence” (Mdn 2.80 versus 3.00) as less of an influence compared to females (U = 19928.0, *p* = 0.044; and U = 21395.0, *p* = 0.001 respectively). 

**Figure 1:**
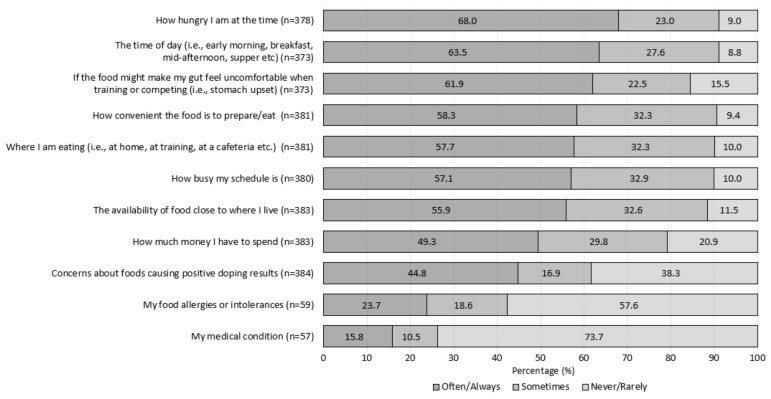
Figure: the percentage of athletes (*n* = 385), who rated how frequently (never/rarely, sometimes and often/always) 11 items influence their food choices.

Ratings for the 11 additional items are presented in Figure 1. There was a high proportion of athletes (*n* = 315, 84.5%) who often or always considered gut comfort when making food choices, with more than half of the participants from each sport category (team *n* = 77, 58%; skill–*n* = 25, 61%; power/sprint *n* = 37, 62%; weight category/aesthetic *n* = 40, 64%; endurance *n* = 30, 68%; and racquet *n* = 21, 70%). Respondents that often/always considered ‘how much money I have to spend’ were younger than those who never/rarely considered this factor influencing food choice (Mdn = 23 (21-26) versus 25 (22-30) years, X^2^ (2) = 8.423, *p* = 0.015). The item, concern for positive doping, received mixed responses (never/rarely, *n* = 147, 38%; often/always *n* = 172, 45%). More athletes from non-Western countries reported often/always being influenced by doping concerns (*n* = 119, 69%), compared to their Western counterparts (*n* = 53, 31%; X^2^ (2) = 14.71, *p* = 0.001). Across the 11 additional items examined, no differences were detected across gender, sport type or event.

A total of 77 comments were received to the open-ended questions on additional factors that may influence food choice. Of these, 42 were removed, as they were represented in the AFCQ or the additional 11 items, and 16 were unrelated to the question. The remaining 19 responses were classified into six categories (Table 3), with the most common response being for ‘preference’.

## 4. Discussion

This study investigated the determinants of food choices in a diverse cohort of athletes and identified differences based on their competition event, sporting type and region. We found that performance was rated highest of all nine factors, and above sensory appeal. While this is in contrast to the literature on general populations that report sensory factors such as taste as one of the highest-ranking influences on food choice [24], previous research in similar competition environments has demonstrated similar outcomes [9]. Yet, the importance of performance influencing food choice may be relevant to the competition event, as we found those competing at the Commonwealth Games were more likely to rank “performance” as well as “food and health awareness” higher than those competing at the Universiade. They also reported being less influenced by others around them and their own emotions when making food choices. Comparing the samples, the Commonwealth Games’ athletes were older and more likely to have been to the Olympic Games, suggesting that they may have been more experienced and successful in their sporting careers. Previous studies have shown more successful athletes to be more emotionally stable than less successful individuals [25]. Consequently, previous experience in the high-pressure competitive environment and being more familiar with the dining hall food environment may mean better control of their emotional state and more confidence making independent food decisions. Similarly, college hockey and American football players have reported that younger players often looked to more senior players to guide their food choices, but less so as they became more experienced [6,8]. The lower ratings for “performance” and “food and health awareness” among Universiade athletes may be attributable to poor nutrition knowledge and the ability to put knowledge consistently into practice [15] 

Other differences in factor ratings were found in relation to sport. “food and health awareness”, “nutritional attributes of the food” and “weight control” influenced food choice in a higher number of athletes from weight category sports compared to others. Comparable findings for the influence of nutrient composition by weight category athletes have been previously reported [9], which is not surprising, considering the stakes that weight has on the athlete’s eligibility to compete. “Usual eating practices” (or familiarity) was considered of similar importance across sports. Interestingly, skill-based athletes considered “usual eating practices” as equally as frequent of an influence on food choices as “performance” and “sensory appeal”. Familiarity has previously been reported as important to athlete food selection at past Commonwealth Games [9], with females more likely to rate the importance of familiarity higher than males. This, in part, may explain our present findings, since skill sport had a higher proportion of female. 

Differences in ratings were also found between athletes from western and non-western countries. For example, “food values and beliefs” was rated as a more frequent influence on food choice by those from non-western countries, which may be attributed to stronger cultural or religious beliefs regarding food. Similarly, a study investigating cultural differences in dietary intake, showed that athletes from Africa, India, Sri Lanka, South East Asia and the Pacific Islands were more likely to report following a diet based on religion than athletes from western regions [26]. 

The phase of training and competition has been shown to have an influence on food choices [6,9]. We found that factors related to nutrition appear to be moderated by competition phase. “Food and health awareness” and “nutritional attributes of the food” were more frequently reported by athletes completing the questionnaire before finishing their competition. The open-ended comments supported this finding, as they made reference to situations relevant to the competition phase such as the “off-season”, “competition day eating” and “weight changes before and between events”. In previous studies, athletes frequently identified competition and training as reasons for their food choice [1,5,6]. The relative importance of key factors influencing food choices are likely to change during the different phase of the competition/season. 

There were some interesting findings from the 11 additional questions on food choice. In particular, a high proportion of athletes across all sport types reported frequently, considering gut comfort in their food choices. Gut discomfort has also been reported as a significant issue for endurance athletes such as triathletes [5] and ultra-endurance athletes [4]. Recent literature has reported improved gastrointestinal comfort with lower FODMAP (fermentable oligo-, di-, mono-saccharides and polyols) intake in athletes [27,28,29,30], and this may be an emerging consideration by some individuals. There were mixed responses about concerns for positive doping influencing food choice. Variation in responses may be reflective of the sample characteristics and the varying level of knowledge athletes have on doping. Previous research has shown that in a mixed cohort of over 384 athletes, only half were aware of the World Anti- Doping Agency regulations and 12% would risk taking prohibited substances to enhance performance [31]. The influence of doping risk differed across regions, with a higher proportion from non-Western countries reporting this as important, suggesting that they were more concerned about doping risk. Although we hypothesized that the student athletes at the Universiade would be more influenced by the money they had to spend on food, this was not the case. However, younger athletes across both events reported being more influenced by their finances, which could impact their ability to choose the appropriate food for their sport.

The open-ended responses suggested that athletes had an interest in trying new foods and experiencing cultural cuisine when in a different country (exploratory eating), and this was part of the competition experience. Food safety was less of a concern, however this may depend on the food environment and level of trust in the catering.

There are limitations in relation to this observational study. Due to convenience sampling, demographic characteristics were not equally distributed across sports and region, and this may have impacted on some results. Nevertheless, our results are supported by the previous literature on this topic.

It is feasible that factors such as performance, cost, convenience and availability would be considered differently by athletes when in their home environment and thus these results are not generalisable to all situations, phases of competition or all athletes. The results were self-reported and as such, there is the potential for social desirability bias and for participants to have been influenced by those around them. Additionally, despite speaking English, this was not the first language for all participants and may have affected the interpretation of the questionnaire.

## 5. Conclusions

This is the first study with a sample of high-performance athletes of this size and diversity that has investigated food choice using a validated tool at two distinct competition events. Performance, sensory appeal and food and health awareness were the most frequently reported influences on food choices of athletes at both events, particularly prior to competition. Commonwealth Games athletes were both more experienced and more influenced by performance and nutrition, and less so by their emotions and other people than Universiade athletes. This suggests that university and college sporting programs could improve education on the impact of nutrition on performance and target key areas that may be adversely influencing athletes’ food choices (for example, coping with emotional eating and healthy eating on a budget). Differences were also found between athletes from different sports and countries that may be reflective of specific performance and cultural nuances.

Future studies on food choice should consider the influence of the food environment, including the cost and accessibility of food, and food safety, where relevant. In general, findings from this study can help athletes and teams better understand the factors that influence their food choices and help inform nutrition education strategies that ultimately improve performance, recovery and the overall health of high-performance athletes.

## Figures and Tables

**Table 1:** Distribution of sex, highest level of previous competition and region (Western or other) across different sport types ^a^ of athletes (*n* = 383).

Characteristic		Total	Weight/aestheti c (*n* = 65) *n* (%)	Power/sprint (*n* = 60) *n* (%)	Endurance (*n* = 45) *n* (%)	Racquet (n = 31) *n* (%)	Team (*n* = 141) *n* (%)	Skill (*n* = 41) *n* (%)	*P* value
**Sex (*n* = 355)**	Male	147	24 (37)	29 (48)	26 (58)	19 (61)	51 (37)	13 (32)	0.014*
	Female	208	41 (63)	31 (52)	19 (42)	12 (38)	88 (63)	28 (68)	
**Highest level of competition (*n* = 353)**	Olympic Games	142	30 (50)	24 (43)	19 (42)	8 (27)	38 (31)	23 (56)	
	World championship	78	10 (17)	13 (23)	11 (24)	6 (21)	27 (22)	11 (27)	
	Continental event ^b^	40	8 (13)	4 (7)	2 (4)	6 (21)	18 (15)	2 (5)	NS
	Universiade	50	4 (7)	7 (13)	5 (11)	3 (10)	27 (22)	4 (10)	
	National competition	43	8 (13)	8 (14)	8 (18)	6 (21)	12 (10)	1 (2)	
**Regions (*n* = 356)**									
	**Western**	**139**	**12 (19)**	**16 (27)**	**20 (44)**	**10 (32)**	**74 (53)**	**21 (51)**	<0.001 *
	Australia, New Zealand	48	3 (6)	12 (24)	4 (8)	5 (10)	20 (39)	7 (14)	
	Canada	63	5 (15)	2 (6)	5 (15)	0 (0)	21 (64)	0 (0)	
	United Kingdom ^c^	28	4 (6)	2 (3)	11 (16)	5 (7)	33 (48)	14 (20)	
	**Other (Non-western)**	**217**	**53 (82)**	**44 (73)**	**25 (56)**	**21 (68)**	**67 (48)**	**20 (49)**	
	Africa	72	16 (21)	16 (21)	12 (16)	2 (3)	26 (34)	4 (5)	
	Asia	35	17 (44)	2 (5)	0 (0)	7 (18)	9 (23)	4 (10)	
	Europe/Middle East	43	3 (7)	8 (19)	5 (12)	10 (23)	10 (23)	7 (16)	
	S.America/Pacific Isles/Caribbean	67	17 (24)	18 (25)	8 (11)	2 (3)	22 (31)	5 (7)	

* Chi-squared: sex X^2^ (5)14.31, regions X^2^ (5)29.47. NS: Not significant (*p* > 0.05); ^a^ Skill—archery, fencing, golf, lawn bowls, shooting; Racquet—badminton, squash, table tennis, tennis; Weight Category/Aesthetic—boxing, gymnastics, judo, powerlifting, taekwondo, weightlift, wrestling, wushu; Team—basketball, beach volleyball, hockey, netball, rugby 7’s, soccer, volleyball, water polo; Endurance—athletics 1,500m–10km, marathon, 20km walk, decathlon, half marathon, steeplechase, road/time trial cycling, swimming; ^b^ Continental events examples: European/African Championships, Commonwealth/Mediterranean/South East Asian/Pacific/Pan American Games; ^c^ Combined United Kingdom for Universiade; England, Ireland, Scotland, Wales and nations of the British Isles for Commonwealth Games.

**Table 2:** Food choice factors median score for athletes (*n* = 385) from event, region and sport type.

	Total	Event			Region ^a^			Sport ^b^						
		Univer-siade	Comm. Games	P value	Western	Other	P value	Weight	Power/sprint	Endur-ance	Racquet	Team	Skill	P Value
Factor	*n* = 385	*n* = 153	*n* = 232		*n* = 153	*n* = 232		*n* = 65	*n* = 60	*n* = 45	*n* = 31	*n* = 141	*n* = 41	
Performance	4.33	4.00	4.33	0.012^*^	4.00	4.33	0.032^&^	4.67	4.83	4.67	4.00	4.00	4.00	<0.001^^^
Sensory appeal	3.75	3.75	4.00	NS	4.00	3.75	NS	3.75	3.75	3.75	3.75	4.00	4.00	NS
Food and health awareness	3.75	3.63	4.00	<0.001^*^	3.75	3.75	NS	4.00	3.75	4.00	3.50	3.75	3.75	0.003^^^
Weight control	3.75	3.75	3.50	NS	3.50	3.75	0.004^&^	3.75	3.88	3.75	3.50	3.50	3.25	0.016^^^
Usual eating practices	3.67	3.33	3.67	0.013^*^	3.67	3.67	NS	3.33	3.67	3.67	3.67	3.67	4.00	NS
Nutritional attributes of the food	3.43	3.43	3.43	NS	3.14	3.71	<0.001^&^	3.86	3.71	3.43	3.29	3.29	3.14	<0.001^^^
Emotional influence	3.00	3.20	2.80	0.001^*^	3.00	3.00	NS	2.60	2.80	3.00	3.20	3.00	3.00	NS
Influence of others	2.83	3.00	2.67	0.002^*^	2.67	3.00	0.023^&^	2.67	2.67	2.67	2.67	3.00	2.67	NS
Food values and beliefs	2.33	2.33	2.33	NS	2.00	2.67	<0.001^&^	2.67	2.33	2.50	2.67	2.00	2.33	0.023^^^

* Mann–Whitney U: Performance 20250.0, Food and health awareness 22405.5, Usual eating practices 20251.0, Emotional influence 14273.0, Influence of others 14282.5. ^&^ Mann–Whitney U: Performance 19880.0, Weight control 20846.0, Nutritional attributes of the food 23528.5, Influence of others 19915.5, Food values and beliefs 23330.0. ^Kruskal–Wallis ANOVA: Performance 37.43, Food and health awareness 18.34, Weight control 13.97, Nutritional attributes of the food 25.16, Food values and beliefs 13.09; NS: Not significant (*p* > 0.05); ^a^ Western—Australia, New Zealand, Canada, United Kingdom, British Isles, Ireland; Other—Africa, Asia, Europe, Middle East, South America, Pacific Islands, the Caribbean; ^b^ Skill—archery, fencing, golf, lawn bowls, shooting; Racquet—badminton, squash, table tennis, tennis; Weight Category/Aesthetic—boxing, gymnastics, judo, powerlifting, taekwondo, weightlift, wrestling, wushu; Team—basketball, beach volleyball, hockey, netball, rugby 7’s, soccer, volleyball, water polo; Endurance—athletics 1500m–10km, marathon, 20km walk, decathlon, half marathon, steeplechase, road/time trial cycling, swimming 400m–800m, triathlon; Power/Sprint—athletics 100m–400m, hurdles, discus, shot put, hammer throw, javelin, long jump, triple jump, pole vault, swimming 50m–200m.

**Table 3:** Additional factors that athletes reported as influencing their food choices.

Category	Responses
Preference (*n* = 5)	When there is too much seasoning in food. I think that the food is good. I love eating and also love cooking. I always making my food different creative style and yummy. I am always happy with simple food. I eat what is available where I live and what I like to eat. Moods, how hungry I am on that day and what type of food I am eating.
Exploratory eating (*n* = 4)	Visiting other countries usually influences me to try their national local or famous dishes. Celebrating birthdays or special occasions has an influence on my choices as well. Being an athlete has the most influence on my regular choices or “often” choices but there will be some days that afford a “cheat day”. Wanting to taste other continents food or cooking. Something I enjoy and can only get outside of Fiji. Curiosity about foods that I am not familiar with.
Competition phase (*n* = 4)	Competition day - nervous often (decrease food volume, increase sports drink). Summer time - off season, so eating basically what I want. Highlight: food before and after competition. One of the big factors is the loss or gain weight for everybody before or between the competition.
Weather (*n* = 4)	The temperature of the room I am in and the country I am in. Weather condition, time, the food I am given. The weather e.g., Cold =soup, warm =salad/cold food. Due to my busy schedule, I eat food that is “on-the-go”. Cold weathers result to warm food, and vice versa.
Food safety (*n* = 1)	The comfort of the environment where the food I have is purchased or being stored (condition of the marketplace and the seller’s health permit). “healthy food environment”.
Transport (*n* = 1)	Transport/car, e.g., can I take a massive watermelon on the bus.
